# Does Atrial Fibrillation Increase the Risk of Developing End-stage Renal Disease in Patients with Chronic Kidney Disease? 

**DOI:** 10.7759/cureus.6908

**Published:** 2020-02-07

**Authors:** Kulachanya Suwanwongse, Nehad Shabarek

**Affiliations:** 1 Internal Medicine, Lincoln Medical Center, New York City, USA

**Keywords:** af, atrial fibrillation, ckd, crd, end stage renal disease (esrd), chronic kidney disease, review article

## Abstract

Patients with atrial fibrillation (AF) have elevated risks of developing stroke, heart failure, and myocardial infarction. However, the impact of AF on the progression of chronic kidney disease (CKD) is uncertain. Our review objective is to investigate whether AF increases the risk of developing end-stage renal disease (ESRD) in patients with CKD. On 31 January 2019, a systemic search was performed on the MEDLINE database using the predefined search criteria. Limits included human participants and English-language publications. Studies that evaluated an association of AF and the risk of CKD progression to ESRD were selected. A total of 751 articles were identified. One prospective cohort study was included after screening abstracts from overall retrieved studies based on our inclusion/exclusion criteria, with a total of 3,091 CKD patients and a mean follow-up of 5.9 years. A total of 172 CKD patients developed AF, of which 43 patients later developed ESRD. Of 2,919 CKD patients with no incident AF, 581 patients progressed to ESRD. The rate of ESRD after the development of AF was 11.8/100 person-years compared with 3.4/100 person-years in CKD patients without AF. In conclusion, AF is an independent risk factor for developing ESRD in CKD patients, but more evidence is needed to support this result.

## Introduction and background

**Figure 1:**
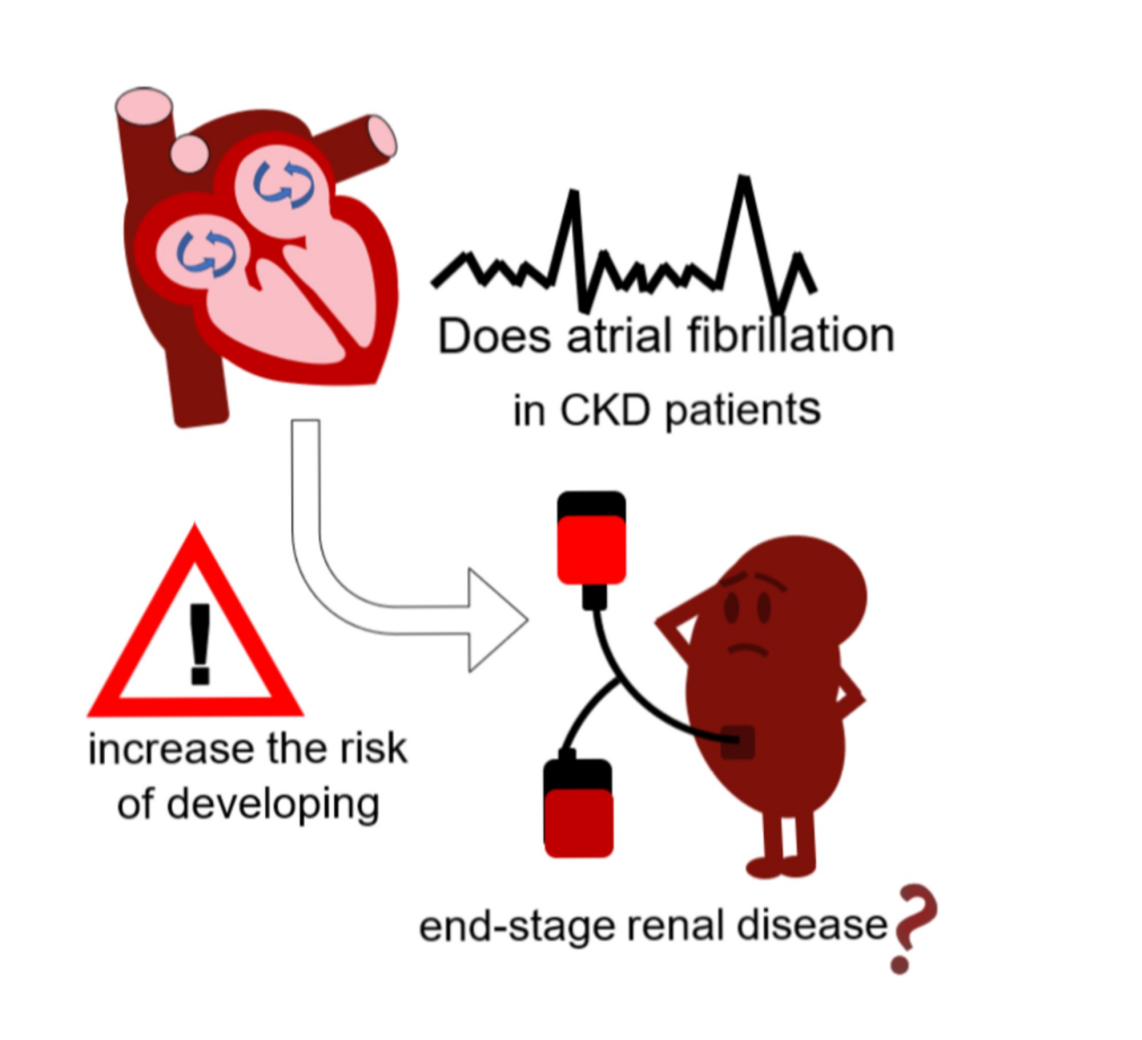
Illustration of an objective of our study CKD, chronic kidney disease.

Atrial fibrillation (AF) is the most common sustained cardiac arrhythmia with more than five million incident cases worldwide [1]. AF significantly impairs the quality of patients' life, and increases patients' mortality, and healthcare burden. Ischemic stroke, heart failure, and myocardial infarction are well-recognized complications of AF [2]. Patients with chronic kidney disease (CKD) have a substantially higher incidence of AF than general population, and AF is a well-known adverse prognostic factor for CKD [3]. However, the impact of AF on the progression of CKD to end-stage renal disease (ESRD) is uncertain. We performed a systemic review to evaluate whether AF aggravated the progression to ESRD in patients with CKD, as demonstrated in Figure 1.

## Review

**Figure 2:**
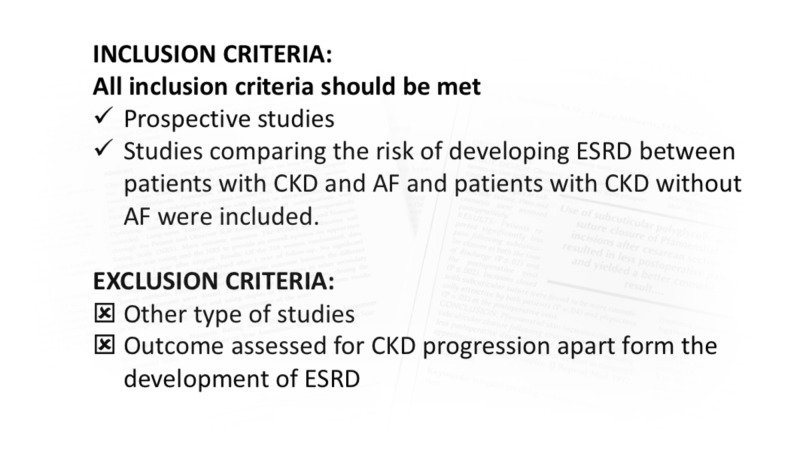
Inclusion/exclusion criteria was applied to select a study from all retrieved abstracts AF, atrial fibrillation; CKD, chronic kidney disease; ESRD, end-stage renal disease.

On 31 January 2019, a systemic search was performed on MEDLINE database using keywords ‘AF’, ‘atrial fibrillation’, ‘CKD’, ‘CRD’, ‘chronic kidney disease’, ‘chronic renal disease’, ‘ESRD’, ‘end-stage renal disease’, ‘end-stage kidney disease’, and ‘dialysis’ from inception to January 2019. Limits included human participants and English-language publications. Studies that evaluated an association of AF and the risk of CKD progression to ESRD were included. Figure 2 demonstrates our inclusion/exclusion criteria. 

**Figure 3:**
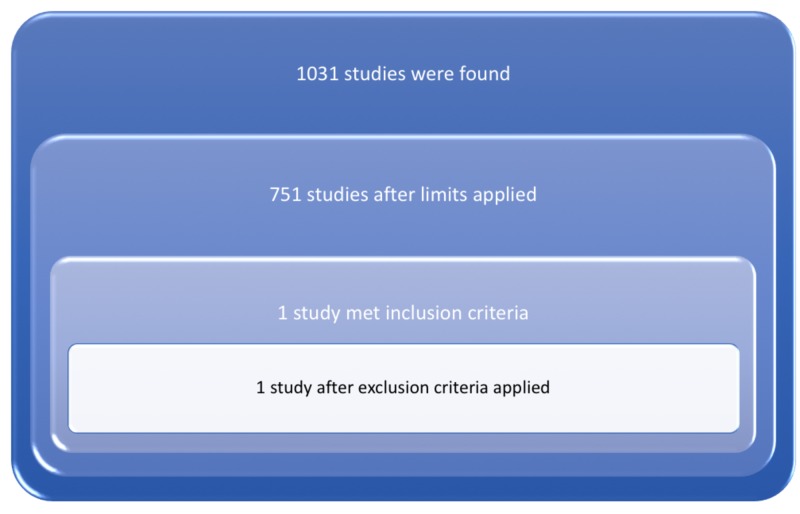
PRISMA (Preferred Reporting Items for Systematic Reviews and Meta-Analyses) for included studies

Our search strategy retrieved a total of 1,031 studies. A total of 751 studies were selected after limits applied. Only one prospective cohort study was identified after screening abstracts from overall retrieved studies based on our inclusion/exclusion criteria, as shown in Figure 3. 

A selected study included a total of 3,091 CKD patients with a mean follow-up of 5.9 years [4]. The research participants enrolled in the Chronic Renal Insufficiency Cohort (CRIC) Study from June 2003 to August 2008 at seven medical centers across the United States. Age-specific estimated glomerular filtration rate (eGFR) criteria were used to define CKD in participants. The study outcome was progression to ESRD, which defined as receipt of chronic dialysis or a kidney transplant from study entry through 31 March 2012 [4].

The result of the study demonstrated that 172 patients with CKD developed AF, of which 43 patients later developed ESRD. Of 2,919 CKD patients with no incident AF, 581 patients progressed to ESRD. The rate of ESRD in CKD patients after the development of AF was 11.8/100 person-years compared with 3.4/100 person-years in CKD patients without the development of incident AF. Multivariable association of incident AF with the risk of ESRD among subgroups with CKD found a strong association across different ranges of age, sex, diabetes status, and baseline eGFR [4].

**Figure 4:**
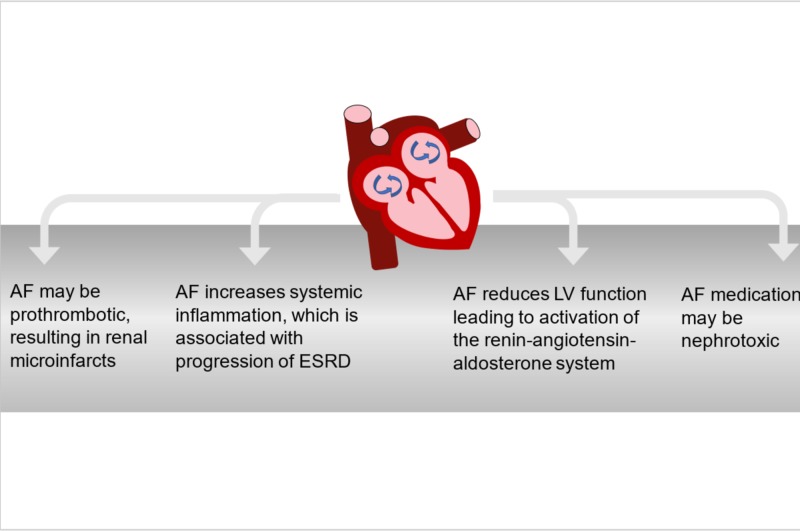
Possible underlying mechanisms that explained the impact of AF on the progression of CKD to ESRD AF, atrial fibrillation; CKD,chronic kidney disease; ESRD, end-stage renal disease; LV,  left ventricular.

The underlying mechanism of an accelerated progression to ESRD in CKD patients with AF remains unclear, which may explain by several possible hypotheses, as summarized in Figure 4. First, AF is a prothrombic state leading to microthrombi occluded renal blood vessels. A decrease in renal perfusion results in renal ischemia and infarction, which in turn impairs kidney function and fastens the progression of CKD to ESRD [5]. Second, AF triggers systemic inflammation, so the kidney functions were declined [6]. Third, AF can lead to a decrease in left ventricular contractility and cardiac output. Therefore, renal perfusion is reduced, resulting in activating the renin-angiotensin-aldosterone system, which further impairs kidney function and accelerated progression to ESRD [7]. Fourth, some medications used in the treatment of AF are nephrotoxic and can result in permanent damage to the kidneys, such as amiodarone. 

Although the study has good experimental designs, which prospectively examined a substantial, diverse sample of well-characterized CKD patients over almost 10 years of follow-up through the CRIC Study, it had several limitations. Overall, the quality of the evidence is low. The first weakness of the study is the method to detect AF, which the authors identified by AF hospitalization with diagnostic codes in the medical record so that some patients who had AF will not be detected. Second, although the authors conducted marginal structural models for the multivariable association of incident AF with the risk of ESRD among subgroups with CKD, it is not comprehensive with lack of several important factors such as smoking status, duration, and severity of diabetes mellitus, and HIV status. Another weakness is the generalizability to the general population as the study was conducted on research volunteers in only one country. Besides, AF itself may lead to more investigations including using intravenous contrast, which can be nephrotoxic [8]. Thus, it may be a confounding factor resulting in CKD progression in the AF group. Lastly, the evidence is based on only one single study so that there is a need to conduct more research evaluating the effect of AF in CKD progression, ideally prospective, multicenter with large sample sizes and addressed all significant multivariable association. 

## Conclusions

AF may be considered an independent risk factor for developing ESRD in patients with CKD. However, more evidence is needed to support this result. Further study should be conducted to identify contributing factors resulting in the accelerated progression to ESRD in CKD patients with AF, which may discover the modifiable risk factors to lower the risk of progression. Patients with CKD and AF may need more aggressive management to prevent morbidity and mortality. 
